# Body Mass Index and In-Hospital Management and Outcomes of Acute Myocardial Infarction

**DOI:** 10.3390/medicina57090926

**Published:** 2021-09-02

**Authors:** Sri Harsha Patlolla, Gayathri Gurumurthy, Pranathi R. Sundaragiri, Wisit Cheungpasitporn, Saraschandra Vallabhajosyula

**Affiliations:** 1 Department of Cardiovascular Surgery, Mayo Clinic, Rochester, MN 55905, USA; Patlolla.SriHarsha@mayo.edu; 2 Division of Pediatric Critical Care Medicine, Department of Pediatrics, Stanford University School of Medicine, Stanford, CA 94305, USA; drgayathri.gurumurthy@gmail.com; 3 Primary Care Internal Medicine, Wake Forest Baptist Health Westwood, High Point, NC 27262, USA; drpranathi99@gmail.com; 4 Division of Nephrology and Hypertension, Department of Medicine, Mayo Clinic, Rochester, MN 55905, USA; wcheungpasitporn@gmail.com; 5 Section of Cardiovascular Medicine, Department of Medicine, Wake Forest University School of Medicine, High Point, NC 27262, USA

**Keywords:** acute myocardial infarction, obesity, cardiovascular risk factors, outcomes research, underweight

## Abstract

*Background and Objectives*: Contemporary data on the prevalence, management and outcomes of acute myocardial infarction (AMI) in relation to body mass index (BMI) are limited. *Materials and Methods*: Using the National Inpatient Sample from 2008 through 2017, we identified adult AMI hospitalizations and categorized them into underweight (BMI < 19.9 kg/m^2^), normal BMI and overweight/obese (BMI > 24.9 kg/m^2^) groups. We evaluated in-hospital mortality, utilization of cardiac procedures and resource utilization among these groups. *Results*: Among 6,089,979 admissions for AMI, 38,070 (0.6%) were underweight, 5,094,721 (83.7%) had normal BMI, and 957,188 (15.7%) were overweight or obese. Over the study period, an increase in the prevalence of AMI was observed in underweight and overweight/obese admissions. Underweight AMI admissions were, on average, older, with higher comorbidity, whereas overweight/obese admissions were younger and had lower comorbidity. In comparison to the normal BMI and overweight/obese categories, significantly lower use of coronary angiography (62.3% vs. 74.6% vs. 37.9%) and PCI (40.8% vs. 47.7% vs. 19.6%) was observed in underweight admissions (all *p* < 0.001). The underweight category was associated with significantly higher in-hospital mortality (10.0% vs. 5.5%; OR 1.23 (95% CI 1.18–1.27), *p* < 0.001), whereas being overweight/obese was associated with significantly lower in-hospital mortality compared to normal BMI admissions (3.1% vs. 5.5%; OR 0.73 (95% CI 0.72–0.74), *p* < 0.001). Underweight AMI admissions had longer lengths of in-hospital stay with frequent discharges to skilled nursing facilities, while overweight/obese admissions had higher hospitalization costs. *Conclusions*: In-hospital management and outcomes of AMI vary by BMI. Underweight status was associated with worse outcomes, whereas the obesity paradox was apparent, with better outcomes for overweight/obese admissions.

## 1. Introduction

The obesity paradox, a hypothesis that obese patients with cardiovascular disease have better outcomes than normal- or low-body-weight patients, has been demonstrated with various cardiovascular diseases, including acute myocardial infarction (AMI) [1,2,3,4]. While various mechanisms have been implicated in the process, there remains uncertainty as to whether the perceived survival advantage for obese patients is truly related to the physiological characteristics associated with excess body weight or due to the aggressive management of these patients [5,6,7]. Since Ellis et al. first described this phenomenon in AMI patients undergoing percutaneous coronary intervention (PCI) [8], several investigators have evaluated the association of body mass index (BMI) with outcomes of cardiovascular diseases, with contrasting findings [2,3,9]. Recent data from the United Kingdom and Australia have shown that the obesity paradox is apparent even in contemporary practice among AMI patients receiving PCI [10,11]. However, contemporary information on whether we still observe this phenomenon with AMI in the United States is unclear and needs to be evaluated, especially due to a steady increase in the AMI admissions of obese and overweight patients over the last decade [12,13]. Regarding the other extreme of BMI, underweight patients or those with low BMI are typically expected to have a greater risk of death due to the presence of cachexia and/or chronic illness [14,15]. Indeed, underweight BMI has been shown to be an independent risk factor for mortality after AMI [15]. Further, it is unclear if the changing demographics and advances in management affected the prevalence of AMI and associated outcomes in recent years across weight categories. Therefore, we assessed the differences in in-hospital events and outcomes of AMI admissions stratified into underweight, overweight/obese and normal BMI.

## 2. Methods

The National (Nationwide) Inpatient Sample (NIS), developed for the Healthcare Quality and Utilization Project (HCUP) through a Federal–State–Industry partnership, is the largest all-payer administrative database of inpatient hospital stays in the United States. The database contains information from a 20% stratified sample of community hospitals [16]. Each discharge record contains information on demographics, hospital characteristics, primary payer, principal and secondary diagnoses and procedures performed during hospitalization. Due to the publicly available nature of this data, we did not request Institutional Review Board approval [16]

The HCUP-NIS data from 1 January 2008 to 31 December 2017 were utilized to identify adult admissions (>18 years) with AMI as the principal diagnosis using International Classification of Diseases Clinical Modification (ICD-CM) codes (ICD-9CM 410.x and ICD-10CM I21.x-22.x) [17,18,19]. Among these AMI admissions, those with ICD codes for BMI < 19.9 kg/m^2^ and/or a diagnosis of underweight were categorized as “underweight”; those with ICD codes for BMI > 24.9 kg/m^2^ and/or a diagnosis of obesity or overweight were grouped into the “overweight/obese” category, and the remaining AMI admissions were considered as “normal BMI”. All administrative codes used in the present study were utilized in the prior literature and are listed in Supplementary Table S1 [20,21]. Comorbid conditions were identified using the Charlson Comorbidity Index based on administrative codes provided by Deyo and colleagues [22]. Baseline, clinical and hospital characteristics, and information on in-hospital procedures, were identified using previous methods (Supplementary Table S1) [17,18,19,23,24,25,26,27,28,29,30,31,32,33,34].

The primary outcome of interest was in-hospital mortality among AMI admissions belonging to each of the three weight categories. The secondary outcomes were use of cardiac procedures such as coronary angiography and PCI, mechanical circulatory support (MCS), length of hospital stay, hospitalization costs and discharge disposition.

### Statistical Analysis

All the pertinent considerations and restrictions of using the HCUP-NIS database were reviewed and addressed [35]. As per the HCUP-NIS recommendations, survey procedures using discharge weights provided with the HCUP-NIS database were used to generate national estimates [35]. Trend weights provided by the HCUP-NIS were used to generate national estimates for samples from 2008–2011 to account for the redesign of the HCUP-NIS in 2012 [35]. Categorical variables were compared using Chi-square tests and reported as percentages. Student *t*-tests or the Mann–Whitney U test were used for continuous variables and these were reported as mean ± standard deviation or median (interquartile range). Trends over time (with 2008 as the referent year) and associations of weight categories with in-hospital mortality were analyzed using multivariable logistic regression. Variables included in the multivariable logistic regression models were age, sex, household median income quartile, primary payer, race, hospital characteristics including location (urban/rural) and teaching status, bedsize and region, comorbidity, cardiac arrest, cardiogenic shock, acute non-cardiac organ failure, use of procedures such as coronary angiography, PCI, MCS, invasive mechanical ventilation and acute hemodialysis. Trends over time in the use of in-hospital cardiac procedures across weight categories were identified. Sensitivity analyses using a multivariable logistic regression with the variables described above were performed in subgroups of age (age ≤ 75 vs. age > 75), sex (male vs. female), type of AMI (STEMI vs. NSTEMI), coronary angiography (yes vs. no) and PCI (yes vs. no) to evaluate associations of weight categories and in-hospital mortality. Purposeful selection of clinically and statistically (liberal threshold of *p* < 0.20 in univariate analysis) relevant variables was conducted for the multivariable regression modeling. All statistical analyses were performed using SPSS v25.0 (IBM Corp, Armonk, NY, USA).

## 3. Results

**Figure 1:**
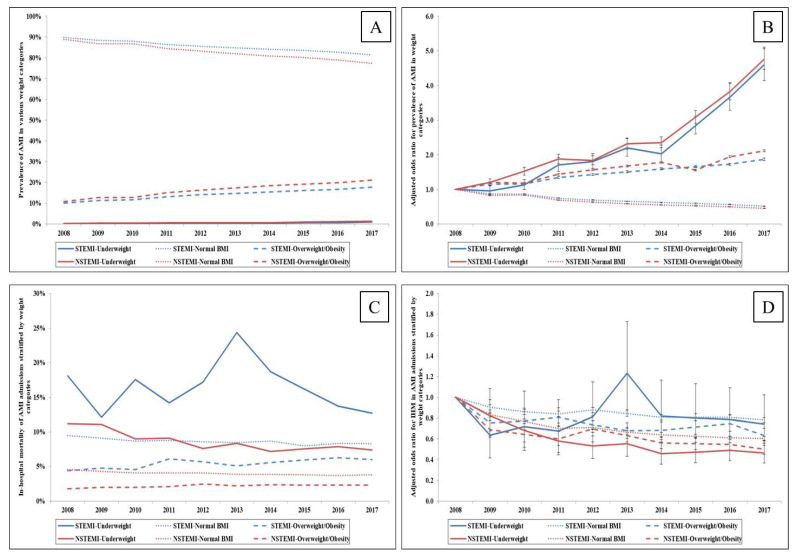
Trends in the prevalence of AMI and in-hospital mortality stratified by weight status. Legend: (**A**): Unadjusted temporal trends of prevalence of AMI across weight categories (*p_trend_* < 0.001); (**B**): Adjusted odds ratio for prevalence of STEMI and NSTEMI in underweight, normal BMI and overweight/obese categories (*p_trend_* < 0.001); (**C**): Unadjusted in-hospital mortality in AMI admissions stratified by weight status and type of AMI (*p_trend_* < 0.001); (**D**): Adjusted odds ratio for in-hospital mortality by year (with 2008 as the referent) in AMI admissions stratified by weight status and type of AMI; (*p_trend_* < 0.001).

Between 1 January 2008 and 31 December 2017, we identified a total of 6,089,979 admissions for AMI, of which 38,070 (0.6%) were underweight, 5,094,721 (83.7%) were grouped as normal BMI and 957,188 (15.7%) were overweight or obese. Underweight and overweight/obesity admissions had a steady increase in AMI prevalence, with a greater prevalence of non-ST-segment-elevation myocardial infarction (NSTEMI) compared to ST-segment-elevation myocardial infarction (STEMI) (Figure 1A). A five-times increase in AMI prevalence was seen in underweight admissions, whereas the prevalence of AMI doubled among overweight/obese admissions in adjusted temporal trends (Figure 1B). Both unadjusted and adjusted analyses revealed a decline in AMI prevalence in normal BMI admissions (Figure 1A,B). Admissions that were underweight were, on average, older, female, of white race, from the lowest median household income quartile and with greater comorbidity when compared to those belonging to normal BMI and overweight/obese categories (Table 1). Compared to normal BMI admissions, overweight/obese patients were younger, more often female and with lower comorbidity scores (Table 1).

**Figure 2:**
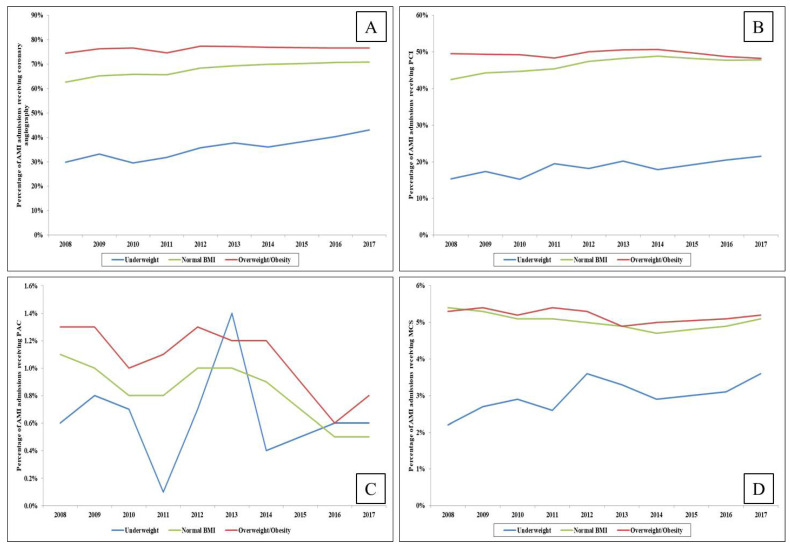
Temporal trends in the use of cardiac procedures in AMI admissions stratified by weight status. Legend: Trends in use of coronary angiography (**A**), percutaneous coronary intervention (**B**), pulmonary artery catheterization (**C**) and mechanical circulatory support (**D**) among AMI admissions stratified by weight categories (*p_trend_* < 0.01 for all).

Underweight and overweight/obese AMI admissions had higher rates of NSTEMI presentation while those with normal BMI had higher rates of STEMI (Table 1). In comparison to those belonging to the normal BMI and overweight/obese categories, underweight admissions had a significantly higher frequency of cardiogenic shock, acute non-cardiac organ failure, lower rates of early coronary angiography, coronary angiography (62.3% vs. 74.6% vs. 37.9%; *p* < 0.001), PCI (40.8% vs. 47.7% vs. 19.6%; *p* < 0.001), coronary artery bypass grafting, MCS and pulmonary artery catheterization (all *p* < 0.001) (Table 1). Temporal trends demonstrated consistently lower use of these procedures in underweight admissions and the highest utilization rates in those who were overweight/obese (Figure 2A–D). Higher rates of invasive and non-invasive mechanical ventilation were seen in underweight admissions in comparison to the other two categories (Table 1).

In the unadjusted analysis, underweight AMI admissions had significantly higher in-hospital mortality (10.0% vs. 5.5% vs. 3.1%, *p* < 0.001) compared to normal BMI AMI admissions and overweight/obese admissions (Table 2). After multivariate logistic regression adjusting for patient and hospital characteristics, comorbidity, cardiac and non-cardiac procedures (Supplementary Table S2), the in-hospital mortality of underweight admissions was higher compared to normal BMI admissions (OR 1.23 (95% CI 1.18–1.27), *p* < 0.001), while significantly lower in-hospital mortality was seen in overweight/obese admissions (OR 0.73 (95% CI 0.72–0.74), *p* < 0.001) compared to AMI admissions with normal BMI. A decline in the in-hospital mortality of underweight and normal BMI admissions, and a slight increase among overweight/obese AMI admissions, was seen in unadjusted temporal trends (Figure 1C). However, adjusted temporal trends showed a decline in in-hospital mortality among all weight categories in both STEMI and NSTEMI admissions (Figure 1D). In further sensitivity analyses, similar findings of higher adjusted in-hospital mortality in underweight AMI admissions and lower adjusted in-hospital mortality in overweight/obese admissions was identified in subgroups of age (age ≤ 75 vs. age > 75), sex (male vs. female), coronary angiography (yes vs. no), PCI (yes vs. no) and those presenting with NSTEMI (Supplementary Table S3). In the subgroup of admissions presenting with STEMI, underweight admissions had in-hospital mortality comparable to normal BMI admissions, whereas overweight/obese admissions had lower in-hospital mortality (Supplementary Table S3). Compared to other categories, those who were underweight had more frequent do-not-resuscitate status, palliative care consultations and longer hospital stay (Table 2). Overweight/obese admissions had higher hospitalization costs and were more likely to be discharged home, while underweight admissions had more frequent discharges to skilled nursing facilities (Table 2).

## 4. Discussion

In this contemporary, nationally representative study, we identified that nearly 16% of AMI admissions in the United States were overweight/obese and 0.6% were underweight. There was an increase in AMI admissions that were underweight or overweight/obese during the study period. Underweight admissions were older, with greater comorbidity, more frequent acute organ failure and had lower rates of angiography, PCI and mechanical circulatory support use, suggestive of a higher burden of frailty. Overweight/obese AMI admissions were younger and more often underwent coronary angiography, PCI and coronary artery bypass grafting. Adjusted in-hospital mortality was higher in the AMI admissions that were underweight, whereas significantly lower in-hospital mortality was identified in overweight/obese AMI admissions in comparison to normal BMI admissions.

In the present study, the incidence of AMI among underweight admissions and overweight/obese admissions increased, consistent with previously reported findings [36]. The association between obesity and coronary atherosclerosis is multifold, with hemodynamic and metabolic factors as well as inflammation and oxidative stress contributing to the development of cardiovascular disease in obese patients [37]. Most overweight/obese AMI admissions from our study were younger and likely female compared to the normal BMI group. In a recent study, Dikaiou et al. showed a significant increase in the risk of AMI from being obese in young women [37]. This furthers the hypothesis that these patients have accelerated atherosclerosis and vulnerable plaque by virtue of their risk factors and inflammatory milieu. On the other hand, underweight AMI admissions from our study were comparatively older, with greater comorbidity. Elderly patients who are underweight have decreased fat stores and physiologic reserve, which may lower their ability to handle acute stress and make them more vulnerable to adverse events, especially in the setting of frailty/cachexia [7,15]. Additionally, the increase in the elderly population in the United States further contributes to the increased prevalence of underweight AMI patients [38]. Nearly 80% of the underweight AMI admissions in our study presented with NSTEMI, which is more frequent among the elderly, consistent with previously reported observations and current population trends [15,39]

Using contemporary data, we identified that the obesity paradox is observed among AMI hospitalizations. Similar to prior studies, overweight/obese AMI admissions were younger, with lower comorbidity and severity of illness, along with a higher utilization of cardiac and non-cardiac procedures [2,3,40]. While some studies have identified the aggressive management of these patients as the reason for the survival benefit [7], others have suggested that excess body fat, increased metabolic reserve and muscle strength confer a protective benefit with respect to tolerating acute stress and higher doses of cardioprotective drugs [6,41]. Further, radial access, which is associated with lower mortality and fewer bleeding events in patients undergoing PCI, is increasingly used in obese/overweight patients and may aid in better prognosis [41]. Bucholz et al. found that obese patients had higher rates of 30-day revascularization, guideline-based therapies on admission and more aggressive secondary prevention after discharge [42]. All these theories possibly explain why overweight/obese patients have had higher use of cardiac procedures, decreased hospital mortality, lower length of hospital stay and improved functional status at discharge compared to the underweight population.

Prior studies have reported that one in five elderly (older than 75 years) AMI admissions are frail [14]. In addition, underweight elderly AMI patients have significantly increased risk of death compared to normal-weight individuals [15]. Consistent with these reports, underweight AMI admissions in the present study, despite having less frequent STEMI presentation than normal BMI and overweight/obese admissions, had greater comorbidity, with higher rates of cardiogenic shock and acute organ failure, contributing to significantly higher in-hospital mortality. Hospitalization of these patients could lead to more weight loss, which can increase their risk of infection and complications, leading to an extended length of stay at the hospital and repeat hospitalizations [15]. Previous reports have shown that underweight patients had several post-procedural complications after PCI, such as hypotension, renal function deterioration, major bleeding, access site hematoma and vascular complications, possibly from excessive anticoagulation [3,43]. This possibly explains why underweight admissions have had higher rates of discharges to skilled nursing facilities in our study. Though these analyses may not be conclusive, studies in the past have shown that older and female patients were comparatively less likely to receive thrombolytics, beta blockers and aspirin after AMI as they have more atypical AMI presentations and hence tend to arrive at the hospital later, thereby limiting their access to these acute cardiac therapies [44,45]. Though these gender differences are not the focus of the study, our study does align with these findings as most of the underweight population in our analysis belonged to the older and female groups. Further, a higher proportion of underweight admissions belonged to lower-income quartiles compared to normal BMI and overweight/obese admissions. Previous studies have demonstrated the influence of socioeconomic inequalities on the management and outcomes of AMI patients, with reportedly lower use of guideline-directed therapies, longer reperfusion times and worse early and late outcomes in those belonging to lower socioeconomic groups [46,47,48]. Damluji and colleagues demonstrated that judicial revascularization in frail older patients with PCI is associated with better survival, while Bucholz et al. have suggested that weight gaining strategies in underweight patients may be beneficial after AMI [14,15].

### Limitations

The present study has several limitations despite the quality control measures used by the HCUP-NIS. The use of previously validated administrative codes for AMI reduces inherent errors associated with coding [17,20]. The lack of granular data, including angiographic, echocardiographic and hemodynamic parameters, prevents the estimation of the severity of disease. Important information on medical management, timing of acute organ failure and other in-hospital events is not available in the database. Use of self-reported height and weight to estimate BMI might have resulted in significant measurement bias, which might have confounded our results. Underreporting of BMI, reliance on administrative codes and misclassification of diagnosis could also have influenced the study results. The database has information only on median household income quartile, and socioeconomic factors beyond this may also have a role in the overall wellbeing of the patients and might have influenced the study outcomes. Residual confounding due to unmeasured confounders could have influenced the observed results. The results of the present study only reflect in-hospital events, and information on post-dismissal outcomes is unavailable.

## 5. Conclusions

Body mass index in AMI appears to influence in-hospital management and outcomes. Underweight status, likely a surrogate of frailty, was associated with worse outcomes, whereas the obesity paradox was apparent in our study, with overweight/obese admissions having lower in-hospital mortality and better outcomes. A better understanding of the sociodemographic–economic factors influencing body weight and their interaction in acute cardiovascular care is crucial to advance the science in this field.

## Figures and Tables

**Table 1:** Baseline and in-hospital characteristics of acute myocardial infarction admissions.

Characteristic		Underweight (*N* = 38,070)	Normal (*N* = 5,094,721)	Overweight/Obese (*N* = 957,188)	*p*
Age (years)		76.7 ± 12.2	68.2 ± 14.1	61.7 ± 12.3	<0.001
Female		62.1	38.2	41.4	<0.001
Race	White	70.6	68.4	68.9	<0.001
	Black	13.1	9.3	11.2	
	Others ^a^	16.3	22.3	19.9	
Primary payer	Medicare	81.9	58.7	46.3	<0.001
	Medicaid	6.2	7.0	9.7	
	Private	8.4	25.3	33.6	
	Others ^b^	3.5	9.0	10.4	
Quartile of median household income for zip code	0–25th	34.0	29.5	30.9	<0.001
	26th–50th	26.7	27.4	27.9	
	51st–75th	21.3	23.6	23.9	
	75th–100th	18.0	19.6	17.3	
Charlson Comorbidity Index	0–3	11.9	37.1	47.4	<0.001
	4–6	48.8	40.9	35.6	
	≥7	39.2	21.9	17.0	
Hypertension		57.6	65.2	72.2	<0.001
Hyperlipidemia		35.5	54.3	64.6	<0.001
Diabetes mellitus, type II		35.5	38.6	40.2	<0.001
Chronic lung disease		37.8	17.8	19.9	<0.001
Moderate/severe kidney disease		21.7	16.4	18.4	<0.001
Cancer		12.6	7.9	4.8	<0.001
Hospital teaching status and location	Rural	10.9	9.7	8.1	<0.001
	Urban non-teaching	32.3	36.3	34.8	
	Urban teaching	56.8	54.0	57.1	
Hospital bed-size	Small	15.2	12.4	12.2	<0.001
	Medium	27.4	26.1	26.8	
	Large	57.3	61.4	61.0	
Hospital region	Northeast	19.4	19.0	15.5	<0.001
	Midwest	23.3	22.7	25.2	
	South	39.5	40.3	40.9	
	West	17.8	18.1	18.5	
Acute myocardial infarction type	ST-segment elevation	19.9	31.3	27.0	<0.001
	Non-ST-segment elevation	80.1	68.7	73.0	
Cardiac arrest		4.7	5.3	4.6	<0.001
Coronary angiography		37.8	67.9	76.5	<0.001
Early coronary angiography (day 0)		14.7	34.7	36.3	<0.001
Percutaneous coronary intervention		19.6	46.5	49.5	<0.001
Coronary artery bypass grafting		4.3	7.9	13.0	<0.001
Cardiogenic shock		6.2	5.7	4.9	<0.001
Acute organ failure	Multi-organ	21.8	12.3	12.1	<0.001
	Respiratory	17.8	10.1	10.6	<0.001
	Hepatic	2.0	1.4	1.2	<0.001
	Renal	23.9	15.6	17.0	<0.001
	Hematologic	7.2	4.6	4.6	<0.001
	Neurologic	10.2	4.2	3.5	<0.001
Mechanical circulatory support		3.0	5.0	5.2	<0.001
Pulmonary artery catheterization		0.7	0.8	1.1	<0.001
Invasive mechanical ventilation		6.8	5.9	6.0	<0.001
Non-invasive mechanical ventilation		4.1	2.1	3.3	<0.001
Acute hemodialysis		0.4	0.6	0.6	<0.001

Legend: Represented as percentage or mean ± standard deviation; ^a^ Hispanic, Asian or Pacific Islander, Native American, Others; ^b^ Self-Pay, No Charge, Others.

**Table 2:** Clinical outcomes of acute myocardial infarction admissions.

Characteristic		Underweight (*N* = 38,070)	Normal (*N* = 5,094,721)	Overweight/Obese (*N* = 957,188)	*p*
In-hospital mortality		10.0	5.5	3.1	<0.001
Length of stay (days)		5 (3–8)	3 (2–5)	3 (2–6)	<0.001
Do-not-resuscitate status		20.4	5.0	2.6	<0.001
Palliative care consultation		10.0	2.3	1.1	<0.001
Hospitalization costs (×1000 USD)		42.9 (22.8–81.8)	51.5 (27.4–88.8)	60.7 (34.6–104.7)	<0.001
Discharge disposition	Home	32.6	64.5	68.6	<0.001
	Transfer	4.6	9.4	9.1	
	Skilled nursing facility	37.9	13.6	10.2	
	Home with home health care	24.0	11.5	11.3	
	Against medical advice	0.9	1.0	0.9	

Legend: Represented as percentage or median (interquartile range). Abbreviations: USD: United States Dollars.

## Data Availability

These data are publicly available for other researchers through the Agency for Healthcare Research and Quality.

## Supplementary Materials

The following are available online at https://www.mdpi.com/article/10.3390/medicina57090926/s1, Table S1: Administrative codes used for identification of diagnoses and procedures. Table S2: Multivariable regression for in-hospital mortality in acute myocardial infarction. Table S3: Sub-group analyses for in-hospital mortality in underweight and overweight/obese AMI admissions compared to normal BMI admissions.
