# Supplementary material for: Body Mass Index and In-Hospital Management and Outcomes of Acute Myocardial Infarction
Source: Medicina (Kaunas). 2021 Sep 2;57(9):926. doi: 10.3390/medicina57090926 (PMC8464976; doi:10.3390/medicina57090926)
Supplement: Supplementary file 1 [file medicina-57-00926-s001.zip › medicina-1360943-supplementary.pdf]

Supplementary Table S1. Administrative codes used for identification of diagnoses and procedures

**Comorbidity**	**International Classification of Diseases 9.0 Clinical Modification Codes**
Acute myocardial infarction	ICD-9CM 410.x and ICD-10CM I21.x-22.x
Underweight	ICD-9CM 783.22, V85.0 and ICD-10CM R63.6, Z68.1
Overweight/obese	ICD-9CM 278.0-278.03, V85.21-V85.45 and ICD-10CM E66.0, E66.01, E66.09, E66.2, E66.3, E66.8, E66.9, Z68.25-Z68.45
Cardiogenic shock	785.51
Cardiac arrest	427.5, 427.4, 427.41, 427.42, 99.60, 99.63
Coronary angiography	37.22, 37.23, 88.53-88.56
Coronary artery bypass grafting	36.10, 36.11, 36.12, 36.13, 36.14, 36.15, 36.16, 36.19
Percutaneous coronary intervention	00.66, 36.01, 36.02, 36.05, 36.06, 36.07, 88.57
Invasive hemodynamic assessment	89.63, 89.64, 89.66, 89.67, 89.68
Mechanical circulatory support	37.61, 37.68, 39.65
Invasive mechanical ventilation	96.7, 96.70, 96.71, 96.72
Hemodialysis	39.95
Multi-organ failure	570.0, 572.2, 573.3, 573.4
	518.81, 518.82, 518.85, 786.09, 799.1, 96.7, 96.70, 96.71, 96.72
	584, 584.5, 584.6, 584.7, 584.8, 584.9
	286.6-286.9, 287.4, 287.5
	293, 293.0, 293.1, 293.8, 293.81-293.84, 293.89, 293.9, 348.1, 348.3, 348.30, 348.81, 348.39, 780.01, 780.09, 89.14

Supplementary Table S2. Multivariable regression for in-hospital mortality in acute myocardial infarction

Total cohort (N = 6,089,979)		Odds ratio	95% confidence interval		p
			Lower Limit	Upper Limit	
Body mass index	Normal		Reference category		
	Underweight	1.23	1.18	1.28	<0.001
	Overweight/Obese	0.73	0.72	0.74	<0.001
Age (years)	≤75 years		Reference category		
	>75 years	2.15	2.13	2.18	<0.001
Female sex		1.15	1.14	1.16	<0.001
Race	White		Reference category		
	Black	0.77	0.76	0.79	<0.001
	Others	1.01	1.00	1.03	0.01
Primary payer	Medicare		Reference category		
	Medicaid	0.73	0.71	0.74	<0.001
	Private	0.67	0.66	0.68	<0.001
	Others	1.02	1.00	1.04	0.04
Quartile of median household income for zip code	0-25th		Reference category		
	26th-50th	0.98	0.96	0.99	<0.001
	51st-75th	0.91	0.90	0.93	<0.001
	75th-100th	0.87	0.86	0.88	<0.001
Hospital teaching status and location	Rural		Reference category		
	Urban non-teaching	0.91	0.89	0.92	<0.001
	Urban teaching	0.96	0.94	0.97	<0.001
Hospital bed-size	Small		Reference category		
	Medium	1.04	1.03	1.06	<0.001
	Large	1.11	1.09	1.12	<0.001
Hospital region	Northeast		Reference category		
	Midwest	0.97	0.95	0.98	<0.001
	South	1.04	1.03	1.06	<0.001
	West	0.89	0.88	0.91	<0.001
Charlson Comorbidity Index	0-3		Reference category		
	4-6	1.75	1.72	1.77	<0.001
	≥ 7	2.03	1.99	2.06	<0.001
Type of acute myocardial infarction	ST-segment elevation		Reference category		
	Non-ST-segment elevation	0.41	0.41	0.41	<0.001
Cardiogenic shock		2.54	2.50	2.57	<0.001
Cardiac arrest		9.99	9.87	10.11	<0.001
Multi-organ failure		3.36	3.32	3.40	<0.001
Coronary angiography		0.38	0.38	0.39	<0.001
Percutaneous coronary intervention		0.39	0.39	0.40	<0.001
Coronary artery bypass grafting		0.36	0.36	0.37	<0.001
Pulmonary artery catheterization		1.10	1.06	1.13	<0.001
Mechanical circulatory support		1.92	1.89	1.95	<0.001
Invasive mechanical ventilation		3.16	3.12	3.20	<0.001
Non-invasive mechanical ventilation		1.45	1.43	1.48	<0.001

Acute hemodialysis	1.62	1.57	1.68	<0.001

Supplementary Table S3. Sub-group analyses for in-hospital mortality in underweight and overweight/obese AMI admissions compared to normal BMI admissions

	**Age ≤75 Years**	**Age >75 Years**
Normal BMI	Reference category	Reference category
Underweight	OR 1.29 [95% CI 1.20-1.39]	OR 1.24 [95% CI 1.18-1.29]
Overweight/Obese	OR 0.79 [95% CI 0.77-0.80]	OR 0.61 [95% CI 0.59-0.63]
	**Male**	**Female**
Normal BMI	Reference category	Reference category
Underweight	OR 1.31 [95% CI 1.23-1.40]	OR 1.22 [95% CI 1.16-1.28]
Overweight/Obese	OR 0.74 [95% CI 0.73-0.76]	OR 0.71 [95% CI 0.69-0.72]
	**ST-segment elevation AMI**	**Non-ST-segment elevation AMI**
Normal BMI	Reference category	Reference category
Underweight	OR 1.02 [95% CI 0.95-1.09]	OR 1.38 [95% CI 1.32-1.45]
Overweight/Obese	OR 0.77 [95% CI 0.76-0.79]	OR 0.69 [95% CI 0.68-0.71]
	**Coronary Angiography**	**No Coronary Angiography**
Normal BMI	Reference category	Reference category
Underweight	OR 1.20 [95% CI 1.10-1.30]	OR 1.29 [95% CI 1.23-1.35]
Overweight/Obese	OR 0.80 [95% CI 0.78-0.81]	OR 0.65 [95% CI 0.63-0.67]
	**PCI**	**No PCI**
Normal BMI	Reference category	Reference category
Underweight	OR 1.22 [95% CI 1.09-1.36]	OR 1.26 [95% CI 1.21-1.32]
Overweight/Obese	OR 0.80 [95% CI 0.78-0.82]	OR 0.69 [95% CI 0.68-0.71]

Legend: The odds ratios reported are multivariable adjusted odds ratios (95% confidence intervals) for in-hospital mortality. Each sub-group was adjusted for age, sex, household median income quartile, primary payer, race, hospital characteristics including location (urban/rural) and teaching status, bedsize, and region, comorbidity, cardiac arrest, cardiogenic shock, acute non-cardiac organ failure, coronary angiography, PCI, MCS, invasive mechanical ventilation, and acute hemodialysis

Abbreviations: AMI: acute myocardial infarction; BMI: body mass index
